# Perceptions and practices of healthcare providers in providing HIV pre-exposure prophylaxis to trans adolescents and young adults and men who have sex with men

**DOI:** 10.11606/s1518-8787.2024058005589

**Published:** 2024-10-11

**Authors:** Ayra Zaine Rodrigues Urbano, Dulce Ferraz, Yzabelle de Lima Raymundo, Eliana Miura Zucchi

**Affiliations:** I Universidade Católica de Santos. Graduation Program in Collective Health. Santos, SP, Brasil Universidade Católica de Santos Graduation Program in Collective Health Santos SP Brasil; II Université Lyon 2. UMR 1296 “Radiations: Defense, Santé, Environnement”. Lyon, França Université Lyon 2 UMR 1296 “Radiations: Defense, Santé, Environnement” Lyon França; III Université de Lausanne. Institut de Psychologie. Lausanne, Suíça Université de Lausanne Institut de Psychologie Lausanne Suíça; IV Fundação Oswaldo Cruz. Escola Fiocruz de Governo. Brasília, DF, Brasil Fundação Oswaldo Cruz Escola Fiocruz de Governo Brasília DF Brasil; V Universidade Católica de Santos. Bachelor’s degree in Nursing. Santos, SP, Brasil Universidade Católica de Santos Bachelor’s degree in Nursing Santos SP Brasil

**Keywords:** Qualitative Research, Healthcare providers, HIV Pre-Exposure Prophylaxis, Care, Adolescents

## Abstract

**OBJECTIVE:**

This study aims to understand the perceptions and practices of healthcare providers regarding the offer of HIV pre-exposure prophylaxis (PrEP) to gay and trans adolescents and young adults.

**METHODS:**

This qualitative research was developed as part of the PrEP1519 study, which was conducted from 2018 to 2021 to analyze the effectiveness of PrEP in adolescents and young adults. Data were collected from July 2020 to February 2021 at the municipality of São Paulo by combining participant observations and semi-structured interviews. The analytical process involved immersion in the empirical material and coding and categorizing it with the support of NVivo®. Interpretation followed the hermeneutic-dialectical principle and had the concept of Care in health practices as its horizon.

**RESULTS:**

The construction of trust-based relationships followed practices that acknowledge the uniqueness of youth and their demands and sought to strengthen their autonomy. Sensitive and supportive listening was pointed out as a welcoming practice that propelled care actions. Welcoming attitudes and support in facing stigma and violence (related or not to the use of PrEP) acknowledged the need to support adolescents and young adults to develop autonomy for prevention. The use of language close to young people’s everyday life favored the construction of relationships of trust and positively influenced the development of autonomy and adherence to PrEP. The tension between technical and practical success occurred in the idealized search for adult-centric normativity as opposed to intersubjectivity.

**CONCLUSION:**

The perceptions and practices of healthcare providers are aligned with the concept of Care as they include actions beyond technical knowledge and recognize the contexts that increase the vulnerability of adolescents and young adults to HIV.

## INTRODUCTION

The growth of the HIV epidemic among adolescents and young adults and the evidence of the effectiveness of HIV pre-exposure prophylaxis (PrEP) in this group led the Brazilian Unified Health System to include its offer for people aged 15 years and over^2^^)^ at the end of 2022. For such a measure to represent a substantial advance, healthcare providers and services must be trained to mitigate access barriers and foster the construction of bonds that ensure confidentiality and acceptance, understanding the health needs of this population based on their life contexts and dimensions of vulnerability to HIV. Thus, understanding the perspectives of both users and healthcare providers becomes imperative in facilitating access to and adherence to PrEP^3^.

The quality of care professionals offer young adults and adolescents depends on several factors. Training professionals to offer PrEP requires expanding technical knowledge on prophylaxis to adequately provide service and follow-up treatment^3^, as well as deconstructing stereotypes and stigmas related to HIV and AIDS, such as the associations between seeking PrEP and promiscuity^4^^), (^^5^, lack of responsibility, and risk compensation^5^^), (^^6^^), (^^7^^), (^^8^^), (^^9^. Additionally, certain characteristics, such as professionals being close in age to the adolescents/young adults, facilitate a better approach^10^. Regarding adherence to PrEP, studies indicate that professionals face challenges in their daily practice including adolescents’ and young adults’ scheduling and adherence to appointments^11^, social and programmatic vulnerabilities that prevent access to the service^12^, and lack of technical training regarding PrEP^13^^), (^^14^. Another issue is that more vulnerable groups still face barriers to access services, such as difficulties in accessing the facilities, inflexible service hours^10^, in addition to homophobia, transphobia, and the stigma related to prevention that sometimes occur in care settings^4^.

Analyzing and understanding the perspective of professionals who assist adolescents and young adults is crucial, as literature indicates that this group’s relationship with health services and their caregivers often involves distrust and fragile bonds, highlighting the importance of consistent follow-up throughout the PrEP use journey and the implementation of additional care strategies^2^^), (^^15^^), (^^16^.

This study aims to understand the perceptions and practices of healthcare providers offering PrEP to trans adolescents and young adults and men who have sex with men (MSM) due to the importance of this method to effectively reducing HIV in these groups.

## METHODS

This qualitative research was conducted as part of the PrEP1519^17^ study, carried out from 2018 to 2021 in São Paulo (in the State of São Paulo), Belo Horizonte (in the state of Minas Gerais), and Salvador (in the state of Bahia). The study aimed to analyze the effectiveness of PrEP among adolescents and young adults. This analysis focused on the municipality of São Paulo, where strategies to create demand for PrEP were implemented in health services, apps, social media, NGOs, and social spaces^18^.

Data were collected from July 2020 to February 2021 and involved semi-structured interviews with healthcare providers who, during the research period, worked with adolescents and young adults using PrEP. Additionally, participant observation of professional team meetings was conducted. Both the interviews and observations were carried out remotely using the Google Meet online meeting platform, which enabled synchronous meetings in response to the sanitary measures adopted to control the COVID-19 pandemic.

The main themes of the interview guide included previous experience with HIV prevention (particularly by PrEP) and with caring for adolescents and young adults; the profile of adolescents and young adults seeking PrEP care; adolescents’ and young adults’ perception regarding the care in the HIV prevention service, the impact of PrEP, and the strategies and weaknesses of the service; barriers and facilitators in access to the service, adherence to care, and PrEP use and adherence. Observations were recorded in field diaries, and the interviews were recorded and fully transcribed.

Data analysis followed the hermeneutic-dialectical principle^19^. After intensive and thorough reading of the transcripts and field diary, data relevance was identified through similarities and contradictions in the narratives. The categories of analysis were developed through an iterative process of induction and deduction using NVivo for support.

The concept of Care^20^ was used as an analytical-interpretative theoretical basis, understood as a normative horizon guiding health practices that exceeds the biomedical dimension by positioning intersubjectivity in the construction and exchange of technical and practical knowledge toward the “happiness project.” This notion, introduced by Ayres^20^, stresses the understanding that must exist in the relationships between providers and young people in the search for the path that sustains and gives meaning to care actions guided by a shared normative horizon. Thus, this study aimed to examine the extent to which the practices of professionals express normative horizons regarding the provision of PrEP to adolescents and young adults, what knowledge is evoked in their daily work, and which elements recognize-or fail to-young people as rights holders in the decision-making process and management of PrEP use.

The consent of all participants was obtained after the informed consent form was read to them. The PrEP1519 study was approved by the Research Ethics Committee of the School of Medicine at Universidade de São Paulo (process 3.082.360).

## RESULTS

This study interviewed eight professionals aged 25 to 52 years: four identified as cis men; three, as cis women; and one as non-binary. Their occupations included three physicians, three psychologists, one biomedical scientist and one nursing technician. Six reported being gay, and two, heterosexual. Four identified as White and four, as Black or of Mixed-race. Four reported having worked with young people; and although all were aware of PrEP, only half had worked with HIV prevention or PrEP before PrEP1519.

### Professionals’ expectations about young PrEP users: balancing judgment and recognizing singularities

A common theme in the participants’ practices was a shift in their positions and expectations regarding adolescents and young adults when faced with their realities. Participants had high expectations that adolescents/young adults would have more knowledge about infection risks and preventive methods and would be diligent and punctual in attending consultations and responsive to follow-up contacts. However, resistance to young people’s behavior also emerged in team interactions. The stigma of adolescence was frequently evident, with a constant desire of adult-like behaviors. Professionals often showed a limited understanding of young people’s choices, leading to conversations that criticized the perceived irresponsibility or lack of commitment of youths.

And it’s really funny, like, because, in my head, I imagine that’s something they already think, right? Even for these subjects, in short, the advancement of the internet, the media, social media as a whole. But when they arrive, it’s often not what goes through their minds, right? [...] So, despite having information about it, right, issues of sexuality, gender, race, sexual orientation, when it comes to sexual practices, is still a very conservative issue (Romeo, counselor).

The perception of young people as more vulnerable required better acknowledgement in relation to creating new knowledge to address specific challenges. Given that young people accessed the service through different strategies that create demand (dating apps, social media, places of sociability, NGOs, and spontaneous demand), their diverse social and programmatic vulnerabilities to HIV resulted in different levels of risk perception, knowledge and interest in PrEP. However, a common theme in the professionals’ narrative regarding vulnerability was the stigma of adolescence, which often conflicted with their expectations of adolescents’ and youth’s autonomy.

I receive them with a little more care because I feel that their vulnerability is much greater. Just like those who are illiterate, when they enter research and have to know everything that happens (Antonela, physician).

Participants highlighted the contrast between their expectations and experience as a need to recognize that each person brings the specificities of their life context to service. Experiences of transphobia in public spaces, adolescents/youths living far from the service, those in family and affective relationships marked by tension and conflict, and the process of learning a care routine were identified as elements requiring specific attention and listening from professionals.

[...] We scheduled an appointment, and it was very difficult for her, because she said: ‘Look, Dante, I just want to schedule it if it’d be late in the afternoon.’ She said: “My fuzz [beard] is growing, and I just can’t... When I shave I get these pink spots, I’m allergic.” We built from that. ‘Look, there’s masks, right?’ [...] And above all it went beyond the service, resuming this conversation of hers, it was precisely ‘the route I need to take to get to (the place of service). What violence can I suffer if they realize that I am not a cis woman? That I’m a trans woman and that I’m here with a beard?’ (Dante, counselor).

Thus, the construction of Care was intrinsically related to recognizing singularities and reorientating professionals’ practice to support the autonomy of adolescents and youths in PrEP use. Given that many young people faced barriers to accessing services, strategies such as financial assistance and transportation aid (e.g., accompanying young people from the subway station to the service) were essential for building trust and fostering connections.

So, for many of them, it’s their first contact with health care, right? That’s why we care for some issues very carefully, certain details, right? So, like, a series of details that maybe an adult person doesn’t need, right? (Valentina, provider working in patient retention).

### The intersubjective dimension of care: building bonds and mobilizing to address the suffering of young adults and adolescents

From the professionals’ point of view, disclosing their sexual orientation (self-declared gay participants) created immediate identification, encouraging young adults and adolescents to share their stories and experiences more openly. Cases were often redistributed among counselors to leverage this identification, as it facilitated the construction of bonds.

Bonding also extended to understanding and sharing guidance on the use of prevention methods (self-testing and PrEP) within the social circles of adolescents and youths.

There were some who became poster boys for prevention and who took condoms, took self-tests to everyone, and advertised PrEP. There were some who shared PrEP with their colleagues. Then, we said: ‘Okay, so come here and I’ll give you another batch because it’s not going to work, you know?’ They started discussing it a lot more and engaged with care. I thought that was cool (Antonela, physician).

Listening to experiences of violence, notably transphobia and homophobia within the family, precarious living conditions, and abusive relationships, intensely mobilized professionals. Many of them had undergone similar experiences at some point in their lives, which deepened their empathy and commitment to providing support.

Abuse is something that sometimes moves me a little, right? So, like, it’s a situation where I try to breathe for a while because I sometimes feel like saying: ‘Boy, stop it.’ You know? Shake them. Because, I don’t know, it could be me, right? Sometimes I want to give the support that I didn’t have. So sometimes, like, I try to be as impersonal as possible, but sometimes I catch myself feeling emotional, you know. Oh fuck, I’m going to cry, sorry. It’s that ‘dad support’ thing, sometimes, you know? So... Or that family thing, you know? So sometimes I catch myself getting emotional. (Matteo, physician).

### The effort toward a horizontal language

An important aspect of professionals’ interactions with adolescents and young adults was learning how to communicate sensitive issues using more colloquial and direct language, “translating” the technical discourse to avoid embarrassing the patients.

So-and-so [referring to a recurring statement by a professional in the service] said: ‘Look, he [referring to the youth] doesn’t know anything, Antonela. You’re going to have to say, ‘you put your dick I don’t know where,’ you know?’ We had to talk like that to some. Then they would widen their eyes like that. So, I said: Yes, that’s just the way it is.’ So, we talk naturally, right? We cuss, and they start laughing. Then they relax and start talking to us, telling us stories [...] And we learn their vocabulary and use it (Antonela, physician).

It is important to highlight the meaning of “he knows nothing” in the previous excerpt as a contrast within the narrative around Care. This expression underscores the need to approach the universe of adolescents and young adults in terms of language and “common sense”. The healthcare providers discourse often reflects an adult-centric perspective that fails to recognize the knowledge and experiences of young people.

Participants reported that young people reacted with surprise when professionals used a repertoire similar to theirs, leading to greater relaxation and openness during consultations. Consequently, the diversity in sexuality and generational characteristics among professionals fostered constructive collaboration, identification, and recognition of the specific life contexts of youths. This approach found a privileged form of expression in the informality of communication, enhancing the effectiveness of care.

And sometimes I, as I’m in it I even try to use slang, right? To see if they’re more comfortable, right? ‘Don’t you do any drugs?’ ‘Nope. None.’ ‘Not even K?’ You know? So, that’s it... Some end up laughing and talking, right? (Matteo, physician).

## DISCUSSION

Acknowledging singularity and fostering a practice focused on learning autonomy in care and prevention are key dimensions professionals prioritize when addressing the vulnerabilities of young adults and adolescents in the context of offering PrEP. This dynamic process evolves continually, aiming to create informed Care practices that treat young adults and adolescents as rights holders. Our findings highlight a range of experiences related to Care and PrEP that converge into practices recognizing each person’s uniqueness within their life context.

The examination of practices reveals a conflict between fulfilling the professional role and the intersubjectivity involved in bonding with patients. Professionals’ expectations, based on an adult-centered view of the knowledge and behavior that adolescents and young adults should have, highlight the ambivalence in their practices. This adult-centric conception reflects how different dimensions of society are organized, normalizing the paradigm of adult behavior and rendering young people invisible as historical subjects and subjects of rights^21^. The programmatic vulnerability affecting them also stems from exclusion based on generational difference. Notions of psychological immaturity and dependence on adults serve to hinder the choices and desires of the present in favor of future success and behaviors that conform to a single model to be followed, i.e. the adult. According to Ayres^22^, Care practices should fully understand the singularities centered on the individual to address each person’s specific needs. However, the pursuit of behavior normativity often results in attempts to standardize young people by having them mirror the behaviors expected of adults. Other studies have noted providers associating behavior deemed responsible and understanding of PrEP efficacy, which leads to identifying young people with a profile more likely to adhere to PrEP. This underscores the importance of recognizing different profiles among young people to provide care tailored to the unique needs of each individual, as line with the concept of Care^20^^), (^^23^^), (^^24^.

Professionals’ expectations about young people’s knowledge about sexual health and prevention can also be seen as an expression of the stigma of adolescence. This stigma manifests in the duality between the marginalization young peoples’ knowledge and expectations while imposing responsibilities aligned with social standards. The recognition of adolescents’ and youths’ vulnerabilities coexists with professionals’ perception of the irresponsibility toward PrEP adherence and service visit frequency. Other studies highlighted the inseparability of the adult-centric construction of adolescents and the processes of stigmatization during care, noting how embarrassment, homophobia, and transphobia can negatively impact adherence to the services and PrEP^22^^), (^^23^^), (^^25^^), (^^26^^), (^^27^^), (^^28^. These studies report that the challenges professionals face when addressing sensitive and/or delicate topics with adolescents - such as mood disorders, substance abuse, and family and affective relationships - further complicate their approach^29^^), (^^30^^), (^^31^.

Convergences were found with studies highlighting the importance of learning of autonomy in developing care actions for young people^32^^), (^^33^. These studies emphasize that the barriers between young people and PrEP can be mediated by a welcoming attitude that addresses demands beyond HIV prevention, recognizing their subjectivity and life context. The perception that a lack of reception drove adolescents and young adults away aligns with findings from a survey conducted with healthcare providers on the care of adolescent and young women using PrEP^3^. This survey noted that care experiences were often mediated by providers’ stigma and discrimination.

Our findings align with studies indicating that care provided by LGBTQIA+ professionals enhances better identification with adolescents and young adults, leading to a perception of greater friendliness within the health service. This creates a context of increased comfort, empathy and trust^34^. Studies with MSM have similarly found that participants reported feeling more comfortable receiving care from gay healthcare providers due to a perceived greater understanding, which aligns more closely with their experiences^24^^), (^^27^^), (^^28^. Discussions about welcoming and supporting personal demands were frequent, reflecting a common reality in HIV prevention practices: the urgency to manage external influences linked to stigma and prejudice regarding preventive choices and sexual practices. These practices can be seen as expressions of Care as they involve the evolution of the capacity for informed decision-making and recognizing the process of the construction of autonomy of adolescents and young people. Autonomy should be understood not only in an individualistic sense but also in its relational dimension, aimed at the collectiveness^35^. This contrasts with literature highlighting adolescents’ and young’ adults perspectives on health services as places of discrimination, transphobia, and stigma and inaccessible language^36^-thus, failing to accommodate the unique ways of life of youths and adolescents. Consequently, practices that enhance identification and support personal demands are fundamental to expand access to the SUS (Brazilian Unified Health System) for socially vulnerable adolescents and young people at higher risk of HIV. Currently, the SUS user profile is predominantly cisgender, white men with higher education^37^.

The reported practices revealed a tension between technical and practical success^22^, highlighting the challenge of balancing active listening and recognizing vulnerabilities with the biomedical rationality that seeks to fulfill stages in Care and ensure high adherence. This approach implicitly assumes control over the behavior of young people to mitigate infection risks.

The use of a less formal language, including slang and dialects typical of the LGBTQIA+ community, was sometimes adopted to better connect with young people. For professionals, this practice constituted a care strategy that facilitated understanding of issues such as drug use and other substances. It also helped establish connections of reciprocal understanding, allowing professionals to identify motivations for adopting safer behaviors regarding HIV. This approach enabled young people to perceive themselves as subjects in their health-related decisions. The choice of a more appropriate language has been discussed in studies showing that it impacts the understanding and trust young people have for the professionals who care for them^38^. Other studies indicate that a lack of effective communication and understanding of the social worlds of adolescents and young adults by professionals creates a significant gap, making it difficult to establish bonds^39^^), (^^40^^), (^^41^. In the programmatic context, young adults and adolescents often face challenges accessing trained and dialogued information in schools. An action integrated with the School Health Program and the Family Health Strategy in Salvador, Bahia, shows the contrast between the idea of school as a privileged locus for sexuality education and the daily structural, social, and programmatic crossings, notably racism, religious conservatism, and the difficulty of joint actions between educators and healthcare providers. Consequently, in the absence of effective family dialogue, young students often rely on peers and the internet for information on sexual and reproductive health^18^. Therefore, providing a welcoming experience in health services requires recognizing the barriers to accessing information in other educational settings.

## FINAL CONSIDERATIONS

PrEP is a strategic prevention technology aimed at reducing new HIV cases among adolescents and young adults. Ensuring this group’s access to PrEP involves implementing welcoming care practices that recognize their subjectivity and promote their autonomy. From the providers’ perspective, fostering young people’s autonomy helps understand their exposure to HIV risk and encourages them to see themselves as active participants in their care. However, the development of autonomy is crucial not just for building adherence to the service but also for moving beyond merely technical success, which often emphasizes criteria like attendance. The effort to construct a more horizontal language helps reduce barriers and stigmas that limit young people’s access to healthcare. This approach strengthens bonding with professionals, aligns expectations and opens opportunities for practical success. These are central dimensions for expanding access to PrEP for those facing greater social vulnerability within the SUS.

The concept of Care articulates the intersubjective dimension of happiness, encompassing what people expect from and wish about life and health. It is essential to look beyond interventions and recognize the importance of the intersubjectivity they entail. The practices examined in this study highlight healthcare providers’ perceptions of reducing the vulnerability of adolescents and youths to HIV. Creating public policies that provide PrEP to adolescents and young adults in public health services has significant challenges, including the role of healthcare providers. As PrEP has made available to adolescents in Brazil only recently, investments are required, particularly in continuous and updated training for the various professionals that can currently prescribe PrEP. These efforts aim to create Care environments that recognize young people’s right to make their own choices and support the development of their autonomy.

### Limitations

Data were collected during the COVID-19 pandemic, which imposed limitations on accessing the study group and conducting follow-up in the work environment, requiring adjustments to traditional observation methods.

This study was part of a project focused on the use of PrEP, which intensifies care for participants and involved selecting and training professionals. This setting differs from the typical public health services that offer HIV preventive methods. This project only considered daily PrEP, opening new horizons for research into various preventive methods, including other modalities of PrEP use (on-demand and injectable).
