# Errata

**DOI:** 10.11606/s1518-8787.2025059005589err

**Published:** 2025-06-27

No artigo “Percepções e práticas de profissionais de saúde na oferta da profilaxia pré-exposição sexual ao HIV para adolescentes e jovens trans e homens que fazem sexo com homens”, DOI http://doi.org/10.11606/s1518-8787.2020054005589, publicado na Revista de Saúde Pública. 2024;58: Suppl 1:10s, RSP corrige:

**Como citar (página 1):**

Onde se lê:

http://doi.org/10.11606/s1518-8787.2020054005589

Leia-se:

http://doi.org/10.11606/s1518-8787.2024058005589

**Rodapé (páginas 1 a 11):**

http://doi.org/10.11606/s1518-8787.2020054005589

Leia-se:

http://doi.org/10.11606/s1518-8787.2024058005589

