# Supplementary material for: Perceptions and practices of healthcare providers in providing HIV pre-exposure prophylaxis to trans adolescents and young adults and men who have sex with men
Source: Rev Saude Publica. 2024 Oct 11;58(Suppl 1):10s. doi: 10.11606/s1518-8787.2024058005589 (PMC11573365; doi:10.11606/s1518-8787.2024058005589)
Supplement: Translation

# Translation: Percepções e práticas de profissionais de saúde na oferta da profilaxia pré-exposição sexual ao HIV para adolescentes e jovens trans e homens que fazem sexo com homens

**Authors:** Ayra Zaine Rodrigues Urbano, Dulce Ferraz, Yzabelle de Lima Raymundo, Eliana Miura Zucchi

**Affiliations:** I Universidade Católica de Santos. Programa de Pós-Graduação em Saúde Coletiva. Santos, SP, Brasil Universidade Católica de Santos Programa de Pós-Graduação em Saúde Coletiva Santos SP Brasil; II Université Lyon 2. UMR 1296 “Radiations: Defense, Santé, Environnement”. Lyon, França Université Lyon 2 UMR 1296 “Radiations: Defense, Santé, Environnement” Lyon França; III Université de Lausanne. Institut de Psychologie. Lausanne, Suíça Université de Lausanne Institut de Psychologie Lausanne Suíça; IV Fundação Oswaldo Cruz. Escola Fiocruz de Governo. Brasília, DF, Brasil Fundação Oswaldo Cruz Escola Fiocruz de Governo Brasília DF Brasil; V Universidade Católica de Santos. Graduação em Enfermagem. Santos, SP, Brasil Universidade Católica de Santos Graduação em Enfermagem Santos SP Brasil

**Keywords:** Pesquisa Qualitativa, Profissionais de Saúde, Profilaxia Pré-Exposição ao HIV, Cuidado, Adolescente

## Abstract

**OBJETIVO:**

Compreender as percepções e práticas de profissionais de saúde no contexto da oferta de profilaxia pré-exposição ao HIV (PrEP) entre adolescentes e jovens gays e trans.

**MÉTODOS:**

Trata-se de pesquisa qualitativa desenvolvida como parte do estudo PrEP1519, realizada entre 2018 e 2021, com o objetivo de analisar a efetividade de PrEP entre adolescentes e jovens. Os dados foram produzidos de julho de 2020 a fevereiro de 2021 no sítio de São Paulo, combinando técnicas de observação-participante e entrevistas semiestruturadas. O processo analítico envolveu imersão no material empírico, codificação e categorização, com apoio do *software* NVivo®. A interpretação seguiu o princípio hermenêutico-dialético e teve como horizonte o conceito de cuidado inserido nas práticas de saúde.

**RESULTADOS:**

A construção do vínculo de confiança foi informada por práticas que reconheciam a singularidade dos/as adolescentes/jovens e suas demandas e buscavam impulsionar sua autonomia. A escuta sensível e solidária foi apontada como uma prática de acolhimento propulsora de ações de cuidado. Atitudes acolhedoras e suporte frente a situações de estigma e violências, relacionadas ou não ao uso de PrEP, convergiram para o reconhecimento da necessidade de apoio no desenvolvimento de autonomia para a prevenção entre adolescentes e jovens. O uso de linguagem próxima do cotidiano favoreceu a construção de relações de confiança, influenciando positivamente o desenvolvimento da autonomia e a adesão a PrEP. A tensão entre êxito técnico e sucesso prático foi observada na busca idealizada pela normatividade adultocêntrica em contraposição à intersubjetividade.

**CONCLUSÃO:**

As percepções e práticas dos/as profissionais de saúde se mostram coerentes com o conceito de cuidado, pois compreendem ações além dos saberes técnicos e reconhecem os contextos que aumentam a vulnerabilidade dos/as adolescentes e jovens ao HIV.

## INTRODUÇÃO

O crescimento da epidemia de HIV entre adolescentes e jovens^1^ e as evidências da efetividade da profilaxia pré-exposição sexual ao HIV (PrEP) para esse grupo levaram, ao final de 2022, à inclusão da oferta desse método para pessoas a partir de 15 anos^2^^)^ no Sistema Único de Saúde. Para que tal medida represente avanço substancial, profissionais e serviços de saúde devem estar qualificados para mitigar barreiras de acesso e fomentar a construção de vínculos que assegurem confidencialidade e acolhimento, compreendendo as necessidades de saúde dessa população a partir de seus contextos de vida e dimensões de vulnerabilidade a infecção por HIV. Assim, a compreensão da perspectiva do/a usuário/a e do/a profissional de saúde mostram-se igualmente relevantes para promover acesso e adesão a PrEP^3^.

A qualidade da atenção ofertada por profissionais para jovens e adolescentes depende de um conjunto de fatores. A capacitação de profissionais para oferecer PrEP requer ampliar não apenas o conhecimento técnico sobre profilaxia para fazer oferta e seguimento adequados^3^, mas também desconstruir estereótipos e estigmas relacionados a HIV e aids que se expressam, por exemplo, nas associações entre busca de PrEP e promiscuidade^4^^), (^^5^, falta de responsabilidade e compensação de risco^5^^), (^^6^^), (^^7^^), (^^8^^), (^^9^. Por outro lado, características dos/as profissionais, como ter idade próxima à do/a adolescente/jovem, facilitam a aproximação^10^. Em relação à adesão a PrEP, estudos indicam que entre os desafios enfrentados por profissionais no cotidiano estão agendamento e comparecimento de adolescentes e jovens às consultas^11^, vulnerabilidades sociais e programáticas que impedem o acesso ao serviço^12^ e falta de capacitação técnica sobre PrEP^13^^), (^^14^. Outro elemento diz respeito à constatação de que segmentos mais vulneráveis ainda encontram barreiras de acesso aos serviços, como dificuldades para chegar e inflexibilidade nos horários de atendimento^10^, além de homofobia, transfobia e estigma relacionado à prevenção, por vezes presentes no atendimento^4^.

Além disso, analisar e compreender a perspectiva do/a profissional que atende adolescentes e jovens é importante se for considerada a literatura que mostra que a relação desse grupo com os serviços de saúde e os/as profissionais que os/as atendem é frequentemente marcada por desconfiança e fragilidade de vínculo, evidenciando a importância do acompanhamento em toda a trajetória do uso de PrEP, bem como das estratégias adicionais de cuidado^2^^), (^^15^^), (^^16^.

O objetivo deste estudo é compreender as percepções e práticas de profissionais de saúde no contexto da oferta de PrEP para adolescentes e jovens trans e homens que fazem sexo com homens (HSH), justificando-se pela importância desse método para a efetiva redução do HIV nesses grupos.

## MÉTODOS

Esta pesquisa qualitativa foi desenvolvida no contexto do estudo PrEP1519^17^, conduzido entre 2018 e 2021 nas cidades de São Paulo (SP), Belo Horizonte (MG) e Salvador (BA), com o objetivo de analisar a efetividade de PrEP entre adolescentes e jovens. A presente análise foi desenvolvida no sítio de São Paulo, onde foram empregadas estratégias de criação de demanda para PrEP em serviços de saúde, aplicativos, redes sociais, ONG e espaços de sociabilidade^18^.

A produção de dados foi realizada entre julho de 2020 e fevereiro de 2021 e envolveu entrevista semiestruturada com profissionais de saúde que atuaram, no período da pesquisa, no atendimento a adolescentes e jovens que usavam PrEP e observação-participante das reuniões de equipe de profissionais. As entrevistas e observações foram realizadas remotamente, utilizando a plataforma de reuniões *on-line* Google Meet, que permitia encontros de forma síncrona, em razão das medidas sanitárias adotadas naquele período para o controle da pandemia da covid-19.

O roteiro de entrevista abordou como temas principais: experiência prévia com prevenção ao HIV e, em particular, por meio de PrEP; experiência prévia com atendimento a adolescentes e jovens; perfil dos/as adolescentes e jovens que buscam atendimento para uso de PrEP; percepção sobre o atendimento de adolescentes e jovens no serviço de prevenção ao HIV; percepção sobre o impacto do uso de PrEP por adolescentes e jovens; barreiras e facilitadores no acesso ao serviço e vinculação ao cuidado; barreiras e facilitadores no uso e adesão a PrEP; e percepções sobre estratégias e fragilidades do serviço. As observações foram registradas em diários de campo, e as entrevistas, gravadas e transcritas integralmente.

A análise dos dados seguiu o princípio hermenêutico-dialético^19^, e, após leitura intensiva e minuciosa das transcrições e do diário de campo, foi identificada a relevância dos dados por meio de semelhanças e contradições presentes nas narrativas. As categorias de análise foram desenvolvidas a partir do processo iterativo de indução e dedução e elaboradas com o apoio do *software* NVivo.

Utilizou-se como base teórica analítico-interpretativa o conceito do cuidado^20^, compreendido como horizonte normativo norteador das práticas de saúde que excede a dimensão biomédica, na medida em que posiciona a intersubjetividade na construção e nas trocas de saberes técnicos e práticos orientados para o “projeto de felicidade”. Esta noção, apresentada por Ayres^20^, enfatiza a compreensão que deve existir nas relações entre profissionais e adolescentes/jovens na busca pelo caminho que sustenta e confere sentido às ações de cuidado orientadas por um horizonte normativo comum. Assim, buscou-se- neste trabalho examinar em que medida as práticas dos/as profissionais expressam diferentes horizontes normativos acerca da oferta de PrEP para adolescentes e jovens, quais saberes são evocados no cotidiano do trabalho e, especialmente, quais elementos sinalizam o reconhecimento - ou a ausência deste - de jovens e adolescentes como sujeitos de direito no processo de decisão e manejo do uso de PrEP.

O consentimento de todos os participantes foi gravado após leitura do termo de consentimento livre e esclarecido. O estudo PrEP1519 foi aprovado pelo Comitê de Ética em Pesquisa da Faculdade de Medicina da Universidade de São Paulo (processo 3.082.360).

## ANÁLISE DOS RESULTADOS

Foram entrevistados oito profissionais. Em relação às suas características, quatro participantes se identificaram como homens cis, três mulheres cis e uma não binária, com idades entre 25 e 52 anos. Três eram médicos/as, três psicólogos/as, uma biomédica e um técnico de enfermagem. Quanto à orientação sexual, seis se declararam gays/homossexuais, e duas, heterossexuais. Quatro se declararam brancos/as, e quatro, negros/as ou pardos/as. Quatro profissionais relataram experiência de trabalho prévia com adolescentes/jovens, e, embora todos/as tivessem conhecimento sobre PrEP, somente metade havia trabalhado com prevenção ao HIV ou com uso de PrEP antes do PrEP1519.

### Expectativas dos/as profissionais sobre jovens usuários/as de PrEP: entre o julgamento e o reconhecimento das singularidades

Um ponto comum aos participantes em relação a sua prática foi a mudança de seus posicionamentos e expectativas sobre adolescentes e jovens diante da realidade que estes/as apresentavam. Havia grande expectativa de que os/as adolescentes/jovens teriam mais conhecimento sobre risco de infecção, métodos preventivos e, especialmente na rotina de seguimento, que seriam assíduos/as e pontuais às consultas e retornariam/responderiam aos contatos feitos pelo serviço. Essa resistência quanto ao comportamento dos/as adolescentes/jovens também apareceu nas interações entre as equipes. Foram frequentes observações em que o estigma da adolescência estava presente. A adultização dos comportamentos era constantemente desejada, e a baixa compreensão dos/as profissionais diante das escolhas se refletia nas conversas sobre irresponsabilidade ou não comprometimento dos/as jovens.

E é muito engraçado, assim, porque, na minha cabeça, eu imagino que isso é algo que eles pensam já, né? Até por esses assuntos, enfim, o avanço da internet, das mídias, redes sociais como um todo. Mas, quando eles chegam, muitas vezes não é o que passa pela cabeça deles, né? [...] Assim, apesar de ter informação sobre, né? Questão de sexualidade, gênero, raça, orientação sexual, quando vai pras práticas sexuais, ainda é uma questão muito conservadora (Romeo, vinculador).

A percepção do/a adolescente/jovem enquanto pessoa mais vulnerável foi tida como motivo para se dar maior atenção a conhecimentos e saberes que precisam ser constituídos. Considerando-se que os/as adolescentes/jovens acessaram o serviço por diferentes estratégias de criação de demanda (via aplicativos de encontro, redes sociais, locais de sociabilidade, ONG e demanda espontânea), havia grande diversidade do ponto de vista da vulnerabilidade social e programática ao HIV, o que se refletia em diferentes graus de percepção de risco e de conhecimento e interesse por PrEP. Por outro lado, um ponto comum à narrativa dos/as profissionais em relação a vulnerabilidade foi o atravessamento do estigma da adolescência, que se chocou com a expectativa dos/as profissionais acerca da autonomia de adolescentes/jovens.

Eu atendo com um pouco mais de cuidado assim, porque eu sinto que a vulnerabilidade é muito maior. Assim como quem é analfabeto, quando vai entrar em uma pesquisa, e tem que saber tudo que acontece (Antonela, médica).

Este contraste entre expectativa e experiência foi significado pelos/as participantes como necessidade de reconhecer que cada um/a trazia especificidades do contexto de vida ao serviço. Experiências de transfobia nos espaços públicos, adolescentes/jovens que moravam distante do serviço, que viviam em relacionamentos familiares e afetivos marcados por tensão e conflito e o próprio aprendizado de uma rotina de cuidados foram apontados como elementos que requeriam atenção e escuta específicas do/a profissional.

[...] chegamos pra agendar, e aí pra ela foi muito difícil, porque ela falou assim: ‘Olha, Dante, eu só quero agendar se for pro fim da tarde’. Ela falou: ‘Meu chuchu [pelos da barba] tá crescendo, e eu não consigo... Quando eu uso gilete fica... Eu tenho alergia’. E a gente foi construindo isso. ‘Olha, tem máscara, né?’ [...] E, principalmente, era para além do serviço, retomando essa conversa dela, era justamente ‘O trajeto que eu preciso fazer para chegar no (local do serviço). Quais violências que eu posso sofrer se eles perceberem que eu não sou uma mulher cis? Que eu sou uma mulher trans e que eu tô com aqui com barba?’ (Dante, vinculador).

Assim, a construção do cuidado esteve intrinsecamente relacionada ao reconhecimento das singularidades e à reorientação da prática dos/as profissionais para colaborarem na construção da autonomia dos/as adolescentes e jovens no contexto do uso de PrEP. Considerando que muitos adolescentes/jovens tinham barreiras de acesso ao serviço, estratégias como ajuda de custo e de navegação (por exemplo, acompanhar o/a adolescente/jovem da estação de metrô até o serviço) foram centrais para a vinculação.

Então, pra muitos deles, é o primeiro contato aí com cuidado em saúde, né? Por isso que algumas questões a gente cuida com muito cuidado, alguns detalhes, né? Então, assim, uma série de detalhes que talvez uma pessoa adulta não precise, né? (Valentina, retentora).

### A dimensão intersubjetiva do cuidado: a construção de vínculo e a mobilização diante do sofrimento de jovens e adolescentes

Do ponto de vista dos/as profissionais, a revelação de suas orientações sexuais (participantes autodeclarados/as gays/homossexuais) criava identificação imediata, e, a partir de então, os/as jovens e adolescentes abriam mais seus históricos e experiências. Foi frequente a redistribuição de casos entre vinculadores/as por causa do reconhecimento de que a orientação sexual gerava maior identificação e propiciava construção de vínculo.

O vínculo também se estendeu à compreensão e ao compartilhamento de orientações sobre o uso de insumos de prevenção (autoteste e PrEP) nos círculos sociais dos/as adolescentes e jovens.

Tinha uns que viravam garoto propaganda da prevenção e que levavam camisinha, levavam autoteste pra todo mundo e faziam propaganda de PrEP. Tinha uns que dividiam PrEP com o colega. Que aí a gente falou: ‘Tá bom, então vem cá que eu vou te dar mais um lote. Porque não vai dar, sabe?’ Eles começaram a discutir muito mais e traziam, se engajavam no cuidado. Isso eu achei bacana (Antonela, médica).

A escuta de experiências de violência, notadamente transfobia e homofobia na família, de precariedade das condições de vida de alguns/mas adolescentes/jovens, de relacionamentos abusivos, entre outras, mobilizava intensamente os/as profissionais, os/as quais, por vezes, tinham também passado por tais experiências em algum momento de suas vidas.

Abuso é uma coisa que assim, às vezes, mexe um pouco comigo, né? Então, assim, é uma situação que eu tento respirar umas três vezes. Porque às vezes eu tenho vontade de falar: ‘Menino, para com isso’. Sabe? Dar um chacoalhão. Porque eu não sei, pode ser uma coisa minha mesmo, né? De, às vezes, querer dar um apoio, um apoio que eu não tive. Então, às vezes, assim, eu tento ser o máximo impessoal possível, mas às vezes acabo me pegando assim, sabe? Ai caralho, vou chorar, desculpa. Aquela coisa de apoio de pai, às vezes, sabe? Então... Ou aquela coisa de família, sabe? Então, às vezes, eu acabo me pegando. (Matteo, médico).

### O esforço no sentido da construção de uma linguagem horizontal

Um ponto importante na interação com os/as adolescentes e jovens foi a aprendizagem dos/as profissionais sobre como se comunicar acerca de assuntos que poderiam causar constrangimento utilizando linguagem mais coloquial e direta, “traduzindo” o discurso técnico.

O fulano [referindo-se a uma fala recorrente de algum profissional no serviço] falava: ‘Olha, ele [referindo-se ao jovem] não sabe nada, Antonela. Vai ter que falar bem ‘botou o pinto não sei onde’, sabe?’. Tinha uns que a gente tinha que falar assim. Daí eles arregalavam o olho procê assim. Eu falei: ‘É. Mas é assim mesmo’. E a gente vai falando no natural assim, né? E falava palavrão, eles começavam a rir. Daí se soltavam e começavam a contar as conversas, as histórias [...] E a gente vai aprendendo os vocabulários deles e usando no meio, assim (Antonela, médica).

É importante ressaltar o sentido de “ele não sabe nada” no excerto anterior como um contraste que precisa ser apreendido como parte da narrativa sobre cuidado. Na mesma produção discursiva, coexistem o reconhecimento da necessidade de se aproximar do universo dos/as adolescentes e jovens no plano de linguagem e o “senso comum” do discurso de um/a profissional de saúde que expressa uma leitura adultocêntrica e não reconhece os saberes dos/as adolescentes/jovens.

Com efeito, os/as participantes referiram que os/as adolescentes/jovens reagiam com surpresa quando os/as profissionais apresentavam repertório próximo do deles/as, o que contribuía para maior descontração e abertura nos atendimentos. Nesse sentido, pode-se destacar que a própria diversidade em relação a sexualidade e características geracionais dos/as profissionais resultaram em sinergia, identificação e reconhecimento das especificidades de vida como parte do contexto de cuidado dos/as jovens, encontrando na informalidade da comunicação via privilegiada de expressão.

E às vezes eu, como já tô no meio, na verdade, assim até tento usar as gírias, né? Pra ver se eles ficam mais à vontade, né? ‘Não usa nenhuma droga?’ ‘Não. Nenhuma’. ‘Nem a Keyla? Nem a Gisele?’ Sabe? Então assim, aí acaba... Uns acabam rindo e acabam falando, né? (Matteo, médico).

## DISCUSSÃO

O reconhecimento da singularidade e a prática focada no aprendizado da autonomia e do cuidado para a prevenção são as dimensões pelas quais os/as profissionais se orientam ao lidar com o atravessamento das vulnerabilidades vividas pelos/as jovens e adolescentes no contexto da oferta de PrEP, em um processo dinâmico de construção e reconstrução de práticas de cuidado informadas por jovens e adolescentes como sujeitos de direitos. Nossos achados destacam um conjunto de experiências associadas a cuidado e PrEP que convergem enquanto práticas que reconhecem a singularidade da pessoa em seu contexto de vida.

O exame das práticas evidencia o conflito entre o cumprimento do papel profissional e a intersubjetividade presente na construção das relações de vínculo. A expectativa dos/as profissionais, baseada em uma visão adultocentrada acerca do conhecimento e comportamento que adolescentes e jovens teriam, revela a ambivalência nas práticas realizadas. A concepção adultocêntrica expressa como diferentes dimensões da sociedade se organizam e normatizam o paradigma do comportamento adulto que invisibiliza adolescentes/jovens enquanto sujeitos históricos e de direitos^21^. A vulnerabilidade programática que os/as atinge é também fruto da exclusão baseada em diferença geracional, e se utiliza de marcadores, como a ideia de imaturidade psicológica e dependência em relação a adultos, para veicular modos de apagamento que negam escolhas e desejos do presente em função de um êxito futuro e de comportamentos e escolhas que expressam o padrão de um único modelo - o adulto - a ser seguido. As práticas de cuidado, segundo Ayres^22^, sugerem ações que compreendem integralmente as singularidades, centradas no sujeito, de modo a atenderem as especificidades de cada um/a; em contraposição, a busca pela normatividade de comportamentos e conhecimentos recai na tentativa de padronização do/a adolescente/jovem tendo-o/a por espelho de comportamentos esperados de uma pessoa adulta. A associação entre o comportamento reconhecido pelos/as profissionais como responsável e a compreensão sobre a eficácia de PrEP está presente em outros estudos e suscita a identificação de um perfil de adolescentes/jovens como mais propensos/as ao seguimento de PrEP e reforça a importância de identificar diferentes perfis de adolescentes e jovens para a realização de um atendimento baseado nas necessidades singulares de cada pessoa, conforme propõe o conceito de cuidado^20^^), (^^23^^), (^^24^.

A expectativa dos/as profissionais sobre o conhecimento dos/as jovens e adolescentes sobre saúde sexual e prevenção pode também ser interpretada como expressão do estigma da adolescência que se manifesta na dualidade entre marginalização do saber jovem e expectativas e responsabilidades correspondentes aos padrões sociais. Com o reconhecimento das vulnerabilidades vividas por adolescentes e jovens, coexistia a percepção dos/as profissionais acerca da irresponsabilidade dos/as adolescentes/jovens em relação ao uso de PrEP ou à frequência ao serviço. De modo indissociável à construção adultocêntrica sobre adolescentes, processos de estigmatização presentes no atendimento em busca de PrEP são expressos por outras pesquisas, que relatam situações de constrangimento, homofobia e transfobia, repercutindo, do mesmo modo, quanto essa experiência pode afetar negativamente a vinculação ao serviço e a PrEP^22^^), (^^23^^), (^^25^^), (^^26^^), (^^27^^), (^^28^. Estudos relatam desafios presentes na abordagem por parte dos/as profissionais junto a adolescentes, em relação a temas sensíveis e/ou delicados, como transtornos de humor, uso abusivo de substâncias entorpecentes e relacionamentos familiares e afetivos^29^^), (^^30^^), (^^31^.

Foram encontradas convergências com estudos que abordam a importância da aprendizagem da autonomia no desenvolvimento de ações de cuidado com adolescentes/jovens^32^^), (^^33^, na medida em que as barreiras existentes entre os/as adolescentes/jovens e PrEP são mediadas por uma postura acolhedora a demandas que extrapolam a prevenção ao HIV, reconhecendo sua subjetividade e contexto de vida. A percepção de que a falta de acolhimento afastava adolescentes e jovens é comparável aos achados de uma pesquisa conduzida com profissionais de saúde sobre o atendimento a mulheres adolescentes e jovens para uso de PrEP^3^, que viveram experiências de atendimento mediadas por situações de estigma e discriminação por parte dos/as profissionais.

Nossos achados também são coerentes com estudos que mostram que ser atendido por profissionais identificados/as como pessoas LGBTQIA+ favorece identificação com adolescentes e jovens e reconhecimento de maior amigabilidade do serviço de saúde, conformando um contexto de maior conforto e vínculo de empatia e confiança^34^. Essa dimensão foi identificada em estudos com HSH que relataram maior conforto em serem atendidos por profissionais de saúde gays, pois sentem uma compreensão maior, que os aproxima do entendimento social que vivenciam^24^^), (^^27^^), (^^28^. Falas de acolhimento e suporte a demandas pessoais foram frequentes e revelaram uma realidade comum às práticas de saúde em prevenção ao HIV: a urgência no manejo de influências externas, ligadas a estigma e preconceito acerca de escolhas preventivas e práticas sexuais. Essas práticas podem ser lidas como expressões do cuidado, na medida em que contemplam a evolução da capacidade de tomada de decisões informadas e reconhecem a dimensão processual da construção da autonomia dos/as adolescentes e jovens. Ademais, a autonomia também precisa ser lida de modo a exceder a concepção individualizante, a exemplo da consideração sobre sua dimensão relacional, voltada à coletividade^35^. Este é um importante contraste com a literatura sobre a perspectiva de adolescentes e jovens acerca dos serviços de saúde como locais de discriminação, transfobia, estigma e de linguagem pouco acessível^36^ - portanto, inacessível à singularidade dos modos de vida de jovens e adolescentes. Tais práticas são, consequentemente, fundamentais para a ampliação do acesso ao SUS por adolescentes e jovens em maior vulnerabilidade social ao HIV, onde o perfil de usuários ainda é majoritariamente constituído por homens cisgênero, brancos e com maior escolaridade^37^.

As práticas relatadas demonstram a tensão entre êxito técnico e sucesso prático^22^; a escuta acolhedora e o reconhecimento de vulnerabilidades são também atravessados pela racionalidade biomédica que visa cumprir etapas no atendimento e garantir alta adesão, pressupondo implicitamente ter controle sobre o comportamento dos jovens, o que mitigaria riscos de infecção.

O uso de uma linguagem menos formal e mais próxima ao universo do adolescente/jovem incluía, por vezes, gírias e dialetos próprios da comunidade LGBTQIA+. Para os/as profissionais, essa prática era uma estratégia de cuidado que favorecia o entendimento dos assuntos abordados no atendimento, como consumo de drogas e outras substâncias. Além disso, permitia estabelecer conexões de entendimento recíproco a partir das quais os/as profissionais conseguiam identificar motivações para adoção de comportamentos mais protegidos em relação ao HIV, pois os/as adolescentes/jovens se percebiam sujeitos reconhecidos frente a decisões sobre sua saúde. A escolha por uma linguagem mais próxima e adequada já foi discutida em estudos que evidenciaram que essa construção impacta na compreensão e na confiança dos/as adolescentes/jovens nos/as profissionais que os/as atendem^38^. Em lógica semelhante, outros estudos mostram que a falta de manejo na comunicação e conhecimento dos universos sociais de adolescentes e jovens por parte dos/as profissionais consiste em um abismo que inviabiliza vínculos^39^^), (^^40^^), (^^41^. Ainda no contexto programático, jovens e adolescentes enfrentam desafios de acesso a informação qualificada e dialogada nas escolas. Uma ação integrada ao Programa Saúde na Escola e ao Estratégia Saúde da Família em Salvador, BA, mostra o contraste da ideia de escola como lócus privilegiado para educação em sexualidade e o cotidiano de atravessamentos estruturais, sociais e programáticos, notadamente racismo, conservadorismo religioso e dificuldade de ações conjuntas entre educadores/as e profissionais de saúde. Consequentemente, diante da dificuldade de diálogo na família, jovens escolares têm suas fontes de informação sobre saúde sexual e reprodutiva entre seus pares e na internet^18^. Portanto, propiciar uma experiência acolhedora nos serviços de saúde pressupõe reconhecer barreiras de acesso à informação em outros espaços formativos.

## CONSIDERAÇÕES FINAIS

PrEP é uma tecnologia de prevenção estratégica para a redução de novos casos de HIV entre adolescentes e jovens, por isso é fundamental promover o acesso deste grupo a ela, o que passa por assegurar práticas assistenciais acolhedoras que reconheçam sua subjetividade e promovam sua autonomia. Na perspectiva dos/as profissionais, o exercício da autonomia dos/as adolescentes/jovens favorece o entendimento sobre exposição a risco de infecção por HIV e reverbera na maturidade de reconhecer-se como protagonista de seu cuidado. No entanto, o desenvolvimento da autonomia parece ser mais importante do ponto de vista da capacidade de construir uma relação de adesão ao serviço do que, por vezes, de sinalizar busca por êxito técnico, na medida em que este se apoia em critérios como assiduidade. O esforço pela construção de uma linguagem mais horizontal oportuniza a redução de barreiras e estigmas que limitam o acesso a cuidados em saúde por parte de adolescentes/jovens, fortalecendo o vínculo com os/as profissionais, equalizando expectativas e abrindo espaço para a percepção do sucesso prático. Essas são dimensões centrais para a ampliação do acesso a PrEP por pessoas em situação de maior vulnerabilidade social no âmbito do SUS.

O cuidado dialoga com a dimensão intersubjetiva da felicidade, com o que as pessoas esperam e sonham para a vida e para a saúde. É preciso ver e ir além das intervenções e assumir a importância da intersubjetividade da qual são parte. As práticas aqui relatadas expõem a percepção de profissionais de saúde sobre a redução da vulnerabilidade de adolescentes e jovens ao HIV. A criação de políticas públicas que permitam a oferta de PrEP para adolescentes e jovens nos serviços públicos de saúde apresenta grandes desafios; a atuação dos/as profissionais de saúde é um deles. PrEP para adolescentes é uma realidade recente no Brasil, e assim são necessários novos investimentos, especialmente capacitação permanente e atualizada que contemple as diferentes categorias profissionais que hoje são aptas à prescrição de PrEP, para que haja a criação de ambientes de oferta de cuidado que reconheçam o direito de adolescentes/jovens de realizar suas escolhas e que sejam favoráveis à conquista de sua autonomia.

### Limitações

A produção de dados foi realizada durante a pandemia da covid-19, e o acesso ao grupo estudado, bem como o acompanhamento no ambiente de trabalho, sofreram limitações que implicaram o rearranjo dos métodos tradicionais de observação.

Este estudo foi realizado junto a um projeto demonstrativo do uso de PrEP, que intensifica o cuidado com os/as participantes e realiza a seleção e capacitação de profissionais, realidade diferente da presente nos serviços públicos de saúde que ofertam métodos preventivos ao HIV. Neste projeto foi contemplada somente PrEP de uso diário, o que abre novos horizontes de pesquisa sobre a oferta de diferentes métodos preventivos, incluindo outras modalidades de uso de PrEP (sob demanda e injetável).
