# Supplementary material for: Errata
Source: Rev Saude Publica. 2025 Jun 27;59:e10serr. [Article in Portuguese] doi: 10.11606/s1518-8787.2025059005589err (PMC12207892; doi:10.11606/s1518-8787.2025059005589err)
Supplement: Related texts

# Correction: Erratum

In the article “Perceptions and practices of healthcare providers in providing HIV pre-exposure prophylaxis to trans adolescents and young adults and men who have sex with men”, DOI http://doi.org/10.11606/s1518-8787.2020054005589, published on the Revista de Saúde Pública. 2024;58: Suppl 1:10s, RSP corrects:

**How to cite (page 1):**

Where it reads:

http://doi.org/10.11606/s1518-8787.2020054005589

It should read:

http://doi.org/10.11606/s1518-8787.2024058005589

**Footnote (pages 1 to 11):**

Where it reads:

http://doi.org/10.11606/s1518-8787.2020054005589

It should read:

http://doi.org/10.11606/s1518-8787.2024058005589
